# Toolbox: Creating a systematic database of secretory pathway proteins uncovers new cargo for COPI

**DOI:** 10.1111/tra.12560

**Published:** 2018-04-10

**Authors:** Uri Weill, Eric C. Arakel, Omer Goldmann, Matan Golan, Silvia Chuartzman, Sean Munro, Blanche Schwappach, Maya Schuldiner

**Affiliations:** ^1^ Department of Molecular Genetics Weizmann Institute of Science Rehovot 7610001 Israel; ^2^ Universitätsmedizin Göttingen Institut für Molekularbiologie Humboldtallee 23 D‐37073 Göttingen Germany; ^3^ Max‐Planck Institute for Biophysical Chemistry Göttingen Germany; ^4^ MRC Laboratory of Molecular Biology Cambridge UK

**Keywords:** cargo, COPI, endomembrane, Lam5, SWAT library, yeast

## Abstract

A third of yeast genes encode for proteins that function in the endomembrane system. However, the precise localization for many of these proteins is still uncertain. Here, we visualized a collection of ~500 N‐terminally, green fluorescent protein (GFP), tagged proteins of the yeast Saccharomyces cerevisiae. By co‐localizing them with 7 known markers of endomembrane compartments we determined the localization for over 200 of them. Using this approach, we create a systematic database of the various secretory compartments and identify several new residents. Focusing in, we now suggest that Lam5 resides in contact sites between the endoplasmic reticulum and the late Golgi. Additionally, analysis of interactions between the COPI coat and co‐localizing proteins from our screen identifies a subset of proteins that are COPI‐cargo. In summary, our approach defines the protein roster within each compartment enabling characterization of the physical and functional organization of the endomembrane system and its components.

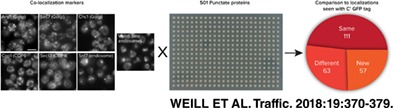

## 1 INTRODUCTION

The secretory pathway is essential for distributing endomembrane proteins to their correct destinations and for the synthesis and targeting of most cellular lipids. Over the years, the repertoire of yeast endomembrane proteins has been studied by a variety of approaches including computational predictions,^1^ functional studies (for few examples see References 2, 3), organelle purification followed by mass spectrometry^4^, ^5^ and by tagging with fluorescent markers.^6^, ^7^ Tagging proteins has the advantage of enabling both determination of cellular localization as well as dynamic tracking of the tagged proteins in living cells.

In the budding yeast, *Saccharomyces cerevisiae* (from here on termed simply yeast), hundreds of secretory proteins were identified through a genome‐wide collection in which each gene was genomically tagged with a green fluorescent protein (GFP) at the C‐terminus (C′).^6^ However, this library fell short of revealing all secretory pathway resident proteins as well as their correct localization for 2 main reasons. First, many proteins in the endomembrane system have a C′ feature required for their correct localization or stabilization, which was obstructed by the C′ tag. Such features include glycosylphosphatidylinositol (GPI) anchors,^8^ tail anchors,^9^ lipid modifications^10^, ^11^ or retrieval motifs.^12^ Second, the native abundance of many proteins is too low to yield an unambiguous GFP signal over the autofluorescence of yeast cells under standard growth conditions. This can be overcome by visualizing the library under a variety of growth conditions. Indeed, by growing yeast in different stress conditions it became possible to identify a handful more of secretory pathway proteins.^13^, ^14^ However, hundreds of predicted endomembrane proteins were still not assigned to any specific compartment and thus could not easily be studied.

To overcome these obstacles in a more systematic way, we have recently constructed a novel yeast collection that includes the majority of secretory proteins genomically tagged with GFP at their N′ (N‐terminus) under the regulation of the constitutive *Saccharomyces paradoxus* (*sp*) NOP1 promoter.^15^ As this library focused on secretory pathway proteins, we used a unique tag for the 316 proteins predicted to include a cleavable signal peptide (SP) at their N′. Such SP containing proteins were tagged with a modified cassette that also included the well‐characterized SP of the Kar2 protein, to allow targeting and insertion of the tagged protein into the endoplasmic reticulum (ER). Altogether, this N′ GFP collection of strains included almost 1800 tagged proteins that were highly expressed, gave a clear GFP signal, and allowed the detection of proteins that are normally not expressed at a high enough abundance to be visualized. In addition, the N′ tag does not interfere with C′ features allowing proper localization of such proteins. Since the ER, plasma membrane (PM) and vacuole have a distinct morphology easily recognized by eye, visual inspection of all strains in this N′ library enabled us to assign localization to these organelles for hundreds of proteins for the first time.^15^

A major hurdle in assigning localization of proteins to secretory compartments is that, at the resolution of a light microscope, many of them appear punctate and indistinguishable from one another. Previously, in the N′ GFP collection, 501 such proteins (130 of which have SPs and were tagged to preserve this feature, and 371 do not) were simply denoted as having “punctate” localization. Here, we use 7 different co‐localization marker proteins of the endomembrane system (for Coat protein complex I (COPI), COPII, Golgi and endosomal compartments) to better assign a defined localization to these proteins. Our systematic co‐localization studies of 3500 different combinations enabled us to determine a localization for over 200 proteins, many of which have not previously been assigned to any organelle. Creating a new secretory protein atlas should help shed light on some of the yet unidentified functions of these secretory compartments.

## 2 RESULTS

### 2.1 Defining protein localization using co‐localization markers

**Figure 1:**
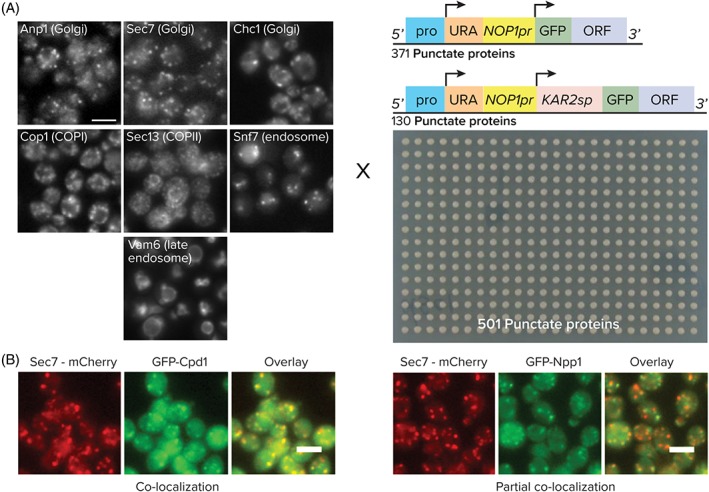
Systematic identification of secretory compartment proteins. A, A collection of 501 strains each expressing an N‐terminally GFP‐tagged protein (expressed under a Nop1 promoter) was crossed with 7 strains each containing a secretory compartment marker tagged with mCherry. B, Examples for 2 possible co‐localization patterns. All scale bars are 5 μm

To assign localization to all punctate N′ GFP‐tagged proteins from the endomembrane library,^15^ we compiled and arrayed 501 yeast proteins that showed a punctate pattern when N′ tagged with GFP (Table S1, Supporting Information). Since 130 of the proteins had a predicted SP they were tagged with a cassette that harbors the Kar2 SP in a way that, following cleavage, the GFP will remain fused to the mature protein (Figure 1A). This array was then crossed with 7 strains of the opposite mating type, each containing a marker protein that resides in one of the major punctate compartments of the secretory pathway or endosomal system, tagged with the red fluorescent protein, mCherry (Figure 1A). Sec13 was used to mark COPII vesicles that carry cargo from the ER to the Golgi apparatus (from hereon termed Golgi); Cop1 was used to define COPI‐coated structures that are classically viewed as intra‐Golgi and retrograde (from the Golgi to the ER) carriers; 3 proteins were used to mark the Golgi—Sec7 and Chc1 that showed a major, but not total, overlap and Anp1 that marked a separate compartment from the other 2 Golgi markers (Figure S1); Snf7 and Vam6 were intended to mark early and late endosomes, respectively (Figure 1A).

The resulting diploids from the cross were imaged with a high content screening setup^13^ based on an epi‐fluorescent microscope. Images were visually scored for the relation between the GFP and mCherry signal patterns. The analysis included 3 possible types of distributions: no co‐localization—where the 2 patterns did not overlap at all; partial co‐localization—where part of the puncta from one channel overlapped with those of the other channel, but not all of them; co‐localization—where the signals from the 2 channels showed a high degree of overlap (Figure 1B). The compilation of these annotations gave the full range of compartment localizations for each N′ GFP‐tagged protein assayed on the one hand, and the full protein roster within each secretory or endosomal compartment on the other (Table S2).

### 2.2 Creating resident protein rosters for secretory and endosomal compartments

Looking at the different co‐localization assignments from our analysis, we set out to ask which protein complexes showed co‐localization to specific compartments (ie, GFP signals co‐localized to the Cherry compartment but not necessarily vice versa). Since the secretory/endomembrane systems are connected by constant flux through vesicular traffic, partial co‐localization during steady state may indicate that a protein is only en route through such a compartment and not a true resident.

In conditions where enzymatic activities of the respective complexes were described in the literature,^16^ this refined localization shed further light on the segregation of reactions by compartmentalization within the endomembrane system. For example, components of the mannosylation machinery were fully co‐localized with the Golgi subcompartment marked by Anp1‐mCherry, which is a subunit of the alpha‐1,6 mannosyltransferase complex (Figure S2). The Golgi subcompartment marked by Sec7‐mCherry, a guanine‐nucleotide exchange factor (GEF) for ADP ribosylation factors involved in vesicular traffic, marks a Golgi subcompartment that indeed included proteins with functions in protein targeting, nucleotide exchange or the exomer complex (Figure S3). Clathrin‐coated membranes visualized by Chc1‐mCherry included proteins with a nucleotide exchange function and components of coat complexes such as Transport protein particle (TRAPP), Chs5p‐Arf1p‐binding proteins (CHAPS) or the clathrin coat itself (Figure S4). Cop1‐marked structures included proteins of the heptameric coatomer complex and proteins with functions in vesicle fusion, nucleotide hydrolysis, and, surprisingly, proteins that take part in the synthesis of phospholipids (Figure S5). Early endosomes included proteins that are involved in phosphatidylinositol 3,5‐bisphosphate binding, membrane traffic or the CORVET complex (Figure S6). Late endosomes and Sec13‐marked structures each had only one protein that fully co‐localized to them—Vti1 and Sec16, respectively (Figure S7). Having only one fully co‐localizing protein may suggest that the markers we chose are not the optimal markers for these compartments or that these compartments are very dynamic or diverse in their protein composition. Another possibility is that yeast does not actually have distinct early and late endosomes as has recently been suggested.^17^

Altogether, this analysis substantially extends the resolution of protein localization and functional segregation within the yeast endomembrane system.

### 2.3 Refined analysis of the COPI‐positive compartment

**Figure 2:**
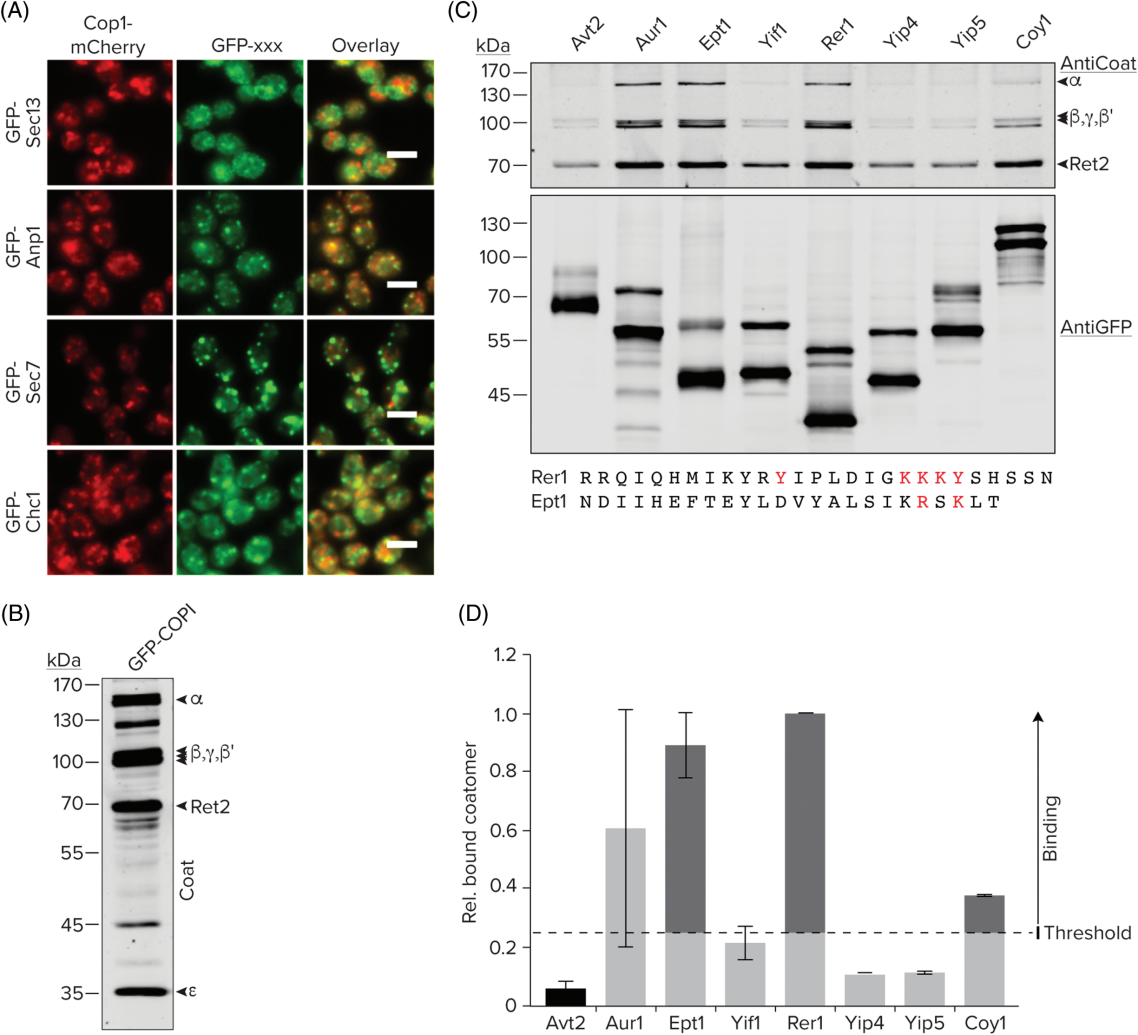
In depth analysis of the COPI compartment. A, Co‐localization assignments for GFP N′ tagged compartment markers of Sec13, Anp1, Sec7 or Chc1 in comparison to the Cop1‐mCherry marker. B, Isolation of intact heptameric coatomer from a GFP‐tagged Cop1/α‐COP strain, demonstrating that the GFP‐tag does not affect complex stability. All western blots were detected with an antibody raised against coatomer. C, Affinity purification of the indicated GFP‐tagged proteins. SDS/PAGE and immunoblot analysis of the eluates using antibodies specific for GFP and coatomer. D, Bar graph of the relative amounts of co‐purified COPI as quantified by the densiometric analysis of immunoblots. Quantification of 2 independent experiments. Error bars depict SEM. Avt2 was chosen as a negative control because it localizes to the vacuole/endosomes. The threshold for non‐specific binding was set at 4 times that of Avt2. All scale bars are 5 μm

To see how our systematic analysis can lend insight into biological processes we focused in on COPI vesicles that are formed by a heptameric coat complex. The alpha‐subunit, Cop1, of the COPI coat was used to ear‐mark COPI‐labeled structures. In support of Cop1 being a viable marker, the COPI‐positive compartment was clearly distinct from COPII‐carrying membranes as marked by Sec13 (Figure 2A). When co‐localizing Cop1‐mCherry with the 3 Golgi markers, we found that it partially co‐localized with all 3 suggesting that COPI is prevalent throughout the Golgi apparatus (Figure 2A). Importantly, subunits of coatomer and several other proteins with verified roles in COPI‐dependent trafficking, such as the Arf GAP Glo3 and members of the COG‐complex,^18^, ^19^ were indeed found to co‐localize with Cop1‐Cherry (Figure S5). And other co‐localizing proteins, such as Eps1, Emp47, Rer1 and Bet1, have been well characterized for their dependence on COPI for their localization.^20^, ^21^, ^22^, ^23^, ^24^, ^25^ In addition, affinity purification of the GFP‐tagged alpha‐subunit (Cop1) resulted in the co‐purification of all 7 subunits of COPI from solubilized membranes (Figure 2B). This result supports the fact that the tag does not perturb complex stability and represents the bona fide localization of COPI in vivo. However, Cop1‐mCherry marked a compartment that was too large to represent a small COPI vesicle with typical dimensions of 70 to 90 nm. This suggests that the compartment marked by our fusion protein was potentially a subdomain of the Golgi apparatus highly active in COPI‐dependent sorting^26^ or represents large carriers such as those observed in the mammalian intermediate compartment.

Despite the important role of the COPI coat in the maintenance of Golgi homeostasis by regulating the transport of lipids and proteins, the extent and hierarchy of its interaction with cargo, adaptors and sorting machinery in vivo is poorly understood. To further characterize which of the proteins that co‐localize with Cop1‐positive structures may interact with COPI, we performed affinity purifications of several such proteins, including Rer1 as a positive control^22^ (Figure 2C). Based on our analysis, we identified 2 coat interactors Ept1 and Coy1. Interestingly, the cytosolic domain of Ept1 contains a putative, poorly characterized RxKxx motif. Previous work demonstrated the retrieval activity of this motif by replacing lysine residues in the KxKxx COPI‐dependent retrieval signal found in many proteins with arginine side chains.^27^ Coy1, has been shown to associate with the COG complex and with intra‐Golgi SNARE proteins.^28^ By demonstrating that Coy1 interacts with COPI we now corroborate an emerging role for Coy1 in intra‐Golgi retrograde transport.

### 2.4 Co‐localization identifies new endomembrane system proteins

**Figure 3:**
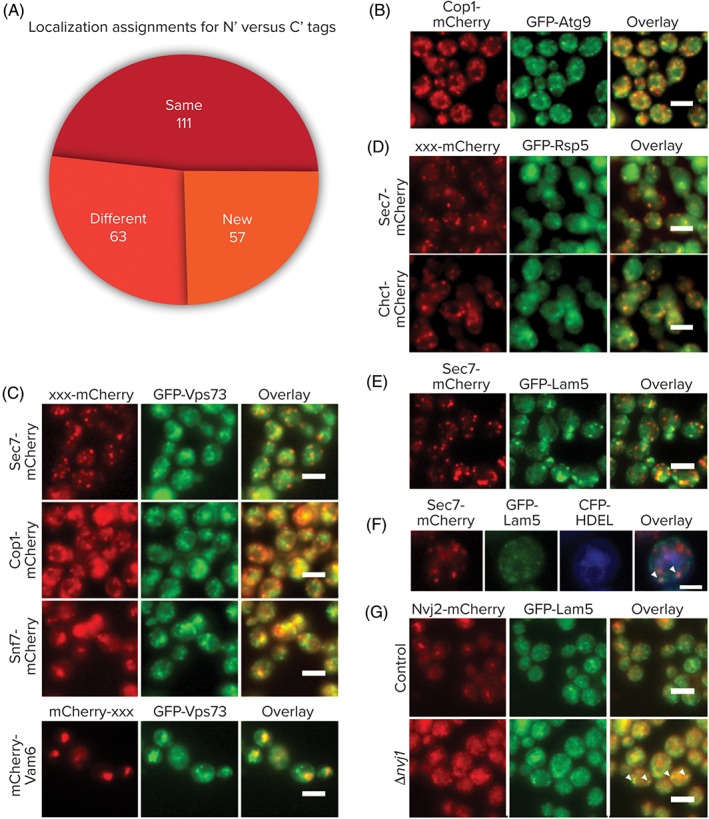
Uncharacterized proteins co‐localizing with each secretory compartment. A, Comparison between the N′ and C′ GFP‐tagged proteins demonstrates a large number of strains that could only be visualized with the N′ tag (New). C‐E, N′ tagged GFP proteins with a defined/suggested function that are co‐localizing with an mCherry tagged marker of secretory compartments. F, Lam5 colocalization with Sec7 occurs in proximity to the ER (HDEL‐CFP). G, Lam5 does not co‐localize with Nvj2 in control cells where it resides at the NVJ, but shows co‐localization in a ∆*nvj1* background when Nvj2 marks ER/Golgi contact sites. All scale bars are 5 μm

Once we assigned a compartment for the analyzed proteins we set out to compare the cellular localizations of the N′ tagged proteins to those same proteins tagged at their C′^6^ (Table S2). Each protein was assigned 1 of 3 categories: “Same”—where both tags show the same localization (110 proteins); “Different”—where each tag had a different defined localization (63 proteins); “New”—where the C′ GFP tag showed a signal below threshold, an ambiguous or no localization but the N′ GFP tag showed a defined cellular localization (57 proteins) (Figure 3A). While many proteins in the “New” category already had well‐defined localizations or known functions from directed and mechanistic work, 10 proteins in this group had neither and our dataset could demonstrate their localization for the first time (Figure S8).

However, some proteins have been previously studied but an accurate localization has not yet been reported for them. An interesting such example is Atg9, the only integral membrane protein involved in forming Cytoplasm to Vacuole (Cvt) and autophagic vesicles.^29^ Atg9 cycles between the phagophore assembly site (PAS) and other cytosolic punctate structures that were previously uncharacterized in yeast. Our analysis now suggests that these punctate structures are, in fact, COPI‐positive (Figure 3B). Atg9 is conserved from yeast to humans^30^, ^31^ and its mammalian homolog, mAtg9, localizes to the trans‐Golgi network and to late endosomes.^32^ The localization to COPI‐positive structures raises the interesting possibility that Atg9 cycles between the endosomal system and the Golgi thereby connecting different compartments recently implicated in autophagosome formation.^33^, ^34^ It has also recently been published that a fraction of COPI binds polyubiquitinated cargo in endosomes for delivery to late‐Golgi.^35^ However, for technical reasons, we could not assay co‐localization of GFP‐Atg9 with the Sec7‐mCherry, Snf7‐mCherry or Vam6‐mCherry markers.

Another illuminating case is Vps73. This gene was originally identified in a screen for vacuolar protein sorting mutants,^36^ however, later it was suggested to reside in mitochondria based on systematic studies of the mitochondrial proteome.^37^ However, our data clearly defines this protein as a vacuolar membrane protein also co‐localizing with COPI vesicles (Cop1), late Golgi (Sec7), early (Snf7) and late (Vam6) endosomes (Figure 3C) putting it in a much more obvious position to affect vacuolar sorting. As it is predicted to be a sugar transporter it may be interesting, in the future, to see whether it affects sugar transport into the Golgi or vacuole.

Rsp5, an E3‐ubiquitin ligase of the NEDD4 family has been previously shown to be involved in regulating multivesicular body (MVB) sorting and endocytosis.^38^ It has also been shown to ubiquitylate Sec23,^39^ a COPII vesicle component and Sna3,^40^ a putative adaptor protein for MVB cargos. Indeed, our N′ tag variant of Rsp5 robustly co‐localizes with both late Golgi markers, Chc1 and Sec7 (Figure 3D), plausibly placing Rsp5 in a region of the Golgi where it can meet its substrates.

Finally, an interesting localization was found for Lam5, one of a family of 6 StART‐domain containing proteins,^41^ whose family members have recently been shown to reside in contact sites and suggested to act in sterol sensing and transfer.^42^ We found that GFP‐Lam5 is not distributed evenly on the ER membrane like a regular membrane protein but rather accumulates in discrete punctate structures on the surface of the ER and is also partially co‐localized with the Sec7‐positive Golgi subcompartment but only in places where it is in proximity to the ER (Figure 3E,F). In support of this, we could experimentally confirm a specific interaction between Lam5 and the Golgi protein Arl1 by co‐immunoprecipitation (data not shown) supporting that Lam5 may be a specific ER/Golgi contact site protein. In order to investigate if Lam5 is located in the newly found, Nvj2 promoted contact sites where ceramide is transferred from the ER to the Golgi,^43^ we checked whether GFP‐Lam5 and Nvj2‐mCherry signals co‐localize (Figure 3E). We found that in control cells where Nvj2 is mostly at the Nuclear Vacuolar Junction, there is no co‐localization between the two. However, in ∆*nvj1* strains where Nvj2 completely relocalizes to the ER/Golgi contact, they do co‐localize. Hence, this is not only proof of Lam5 being a new ER/Golgi contact site protein but also suggests that Lam5 is present at these contacts preceding Nvj2 recruitment and potentially seeding the contact site.

In summary, whether or not they had previously been studied, proteins belonging to the “New” category can now, for the first time, be tracked in live cells and included in imaging screens using our characterized N′ fusion.

### 2.5 N′ tagging warrants a reassessment of the cellular localization of certain proteins

**Figure 4:**
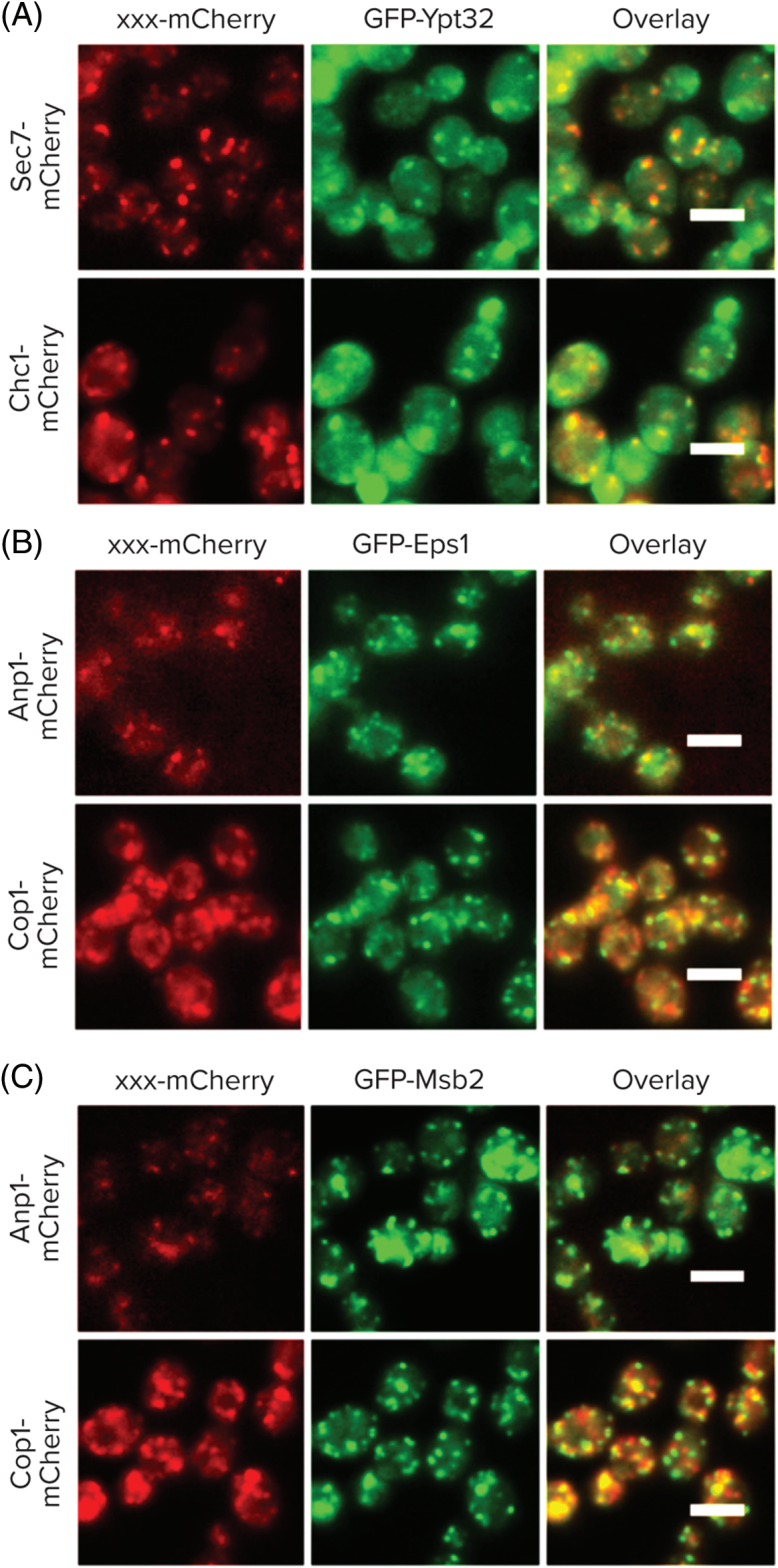
N′ tagging reveals potentially new localizations. Shown are examples of proteins that co‐localized with specific secretory markers (mCherry) only when tagged with GFP at their N′. All scale bars are 5 μm

Another interesting group of proteins that arose from our analysis were those of the “Different” category. Many proteins in this category were well‐characterized proteins of the endomembrane system. However, previous studies had focused on the C′ form that may have been mis‐localized. For example, Ypt32 is a Rab family GTPase that mediates intra‐Golgi traffic or the budding of post‐Golgi vesicles from the trans‐Golgi,^44^, ^45^ when it was tagged at the C′ with GFP it was mis‐localized to the cytosol and the nucleus, but with the N′ GFP tag, Ypt32 correctly co‐localized with the Golgi markers Sec7 and Chc1 (Figure 4A). In the case of Ypt32 and many other proteins of the “Different” group (such as those that have tail anchors, GPI anchors, lipidation sites, C′ retrieval motifs, or those that were previously localized to the cytosol or vacuolar lumen despite having transmembrane domains [TMDs]) it was obvious that the N′ tag showed the correct localization, but for some proteins it is yet to be determined which tag preserves the correct localization of the protein. Potentially, both tagged forms highlight one of the compartments in which the protein resides or both may reflect mis‐localization.

Particularly interesting are proteins annotated as “Different” whose function has not yet been determined: For these uncharacterized proteins, knowledge of their accurate location can give clues as to their cellular function (Figure S9). For example, Gdt1, Uip5, Yil067c, Ykl077w, Ynl115c and Yor292c are uncharacterized proteins that were localized to the cytosol or the vacuole lumen when they were C′ tagged. However, with our N′ tag they all are endomembrane localized. We therefore infer that N′ tagging reveals their correct localization while C′ tagging leads to their mis‐localization. This new information about these uncharacterized proteins may now lead to better understanding of them and their further functional investigation as secretory proteins. For example, we have recently found Ykl077w (which we now call Psg1) to be localized to COPI vesicles and this has enabled us to identify it as an effector of Pma1 trafficking.^46^

There are some cases in which the N′ tag localization warrants a reassessment of a protein's function. For example, Eps1 is a member of the protein‐disulfide isomerase (PDI) family whose 5 members were all previously thought to reside in the ER. Our analysis suggests that this one member, which is unique in having a TMD (all other 4 are soluble enzymes), in fact partially resides in the medial Golgi (Figure 4B) and may function in isomerization of disulfide bonds in this compartment. This interesting because in mammals hQSOX1a, a PDI family member that also has a TMD, is a Golgi resident.^47^, ^48^

Another example is Msb2, a signaling molecule that acts as an osmosensor. While it is known that Msb2 has both a secreted and a cell‐associated form,^49^ we find it co‐localizing with the Golgi marker Anp1 as well as the COPI marker, Cop1 (Figure 4C). While it is possible that our tag slowed down or abolished traffic out of the Golgi, it may also be that it represents a novel destination for Msb2 that could suggest the Golgi as a signaling platform for filamentous growth.

## 3 DISCUSSION

**Figure 5:**
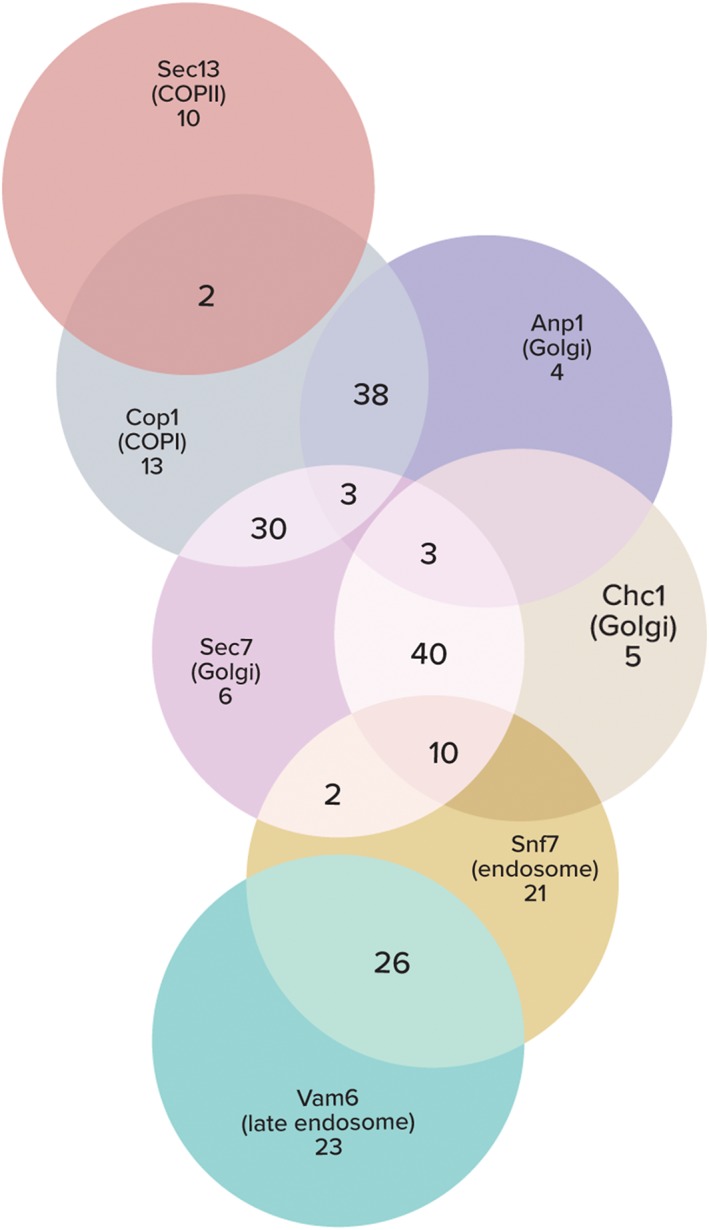
Systematic assignment of subcellular localization reveals a pattern of protein distribution across the secretory system. A Venn diagram of N′ GFP‐tagged proteins localizing to one or more compartments according to full or partial co‐localization with mCherry‐tagged markers. The size of each circle/overlap is not proportional to the number of inhabiting proteins

The aim of our work was to create a systematic atlas of the endomembrane proteome and identify new secretory pathway proteins. To do this we took advantage of the novel collection of N′ GFP‐tagged strains that enables visualization of protein localization for many proteins that until now could not be followed microscopically. An interesting aspect of our analysis was that many proteins co‐localized with more than one compartment marker. This is not surprising as the secretory pathway is interconnected by vesicular traffic and proteins in this system are in constant flux between compartments. Such proteins that are distributed between different endomembrane compartments in steady state could provide interesting information regarding the flow of proteins through the secretory pathway and the extent of overlap in compartment functions (Figure 5). For example, focusing on Golgi subcompartments, it was evident that most proteins that localize to the compartment marked by Sec7 also co‐localize with that marked by Chc1 and that very few proteins from both compartments co‐localize with the Anp1‐labeled compartment. This suggests that, at steady state, most proteins are either in a compartment where mannosylation is occurring (early/medial Golgi) or in transit for secretion (late Golgi). The fact that very little intermixing occurs between early and late Golgi compartments is in agreement with previous studies demonstrating Golgi cisternae maturation.^50^, ^51^ However, Sec7 and Chc1 also had some unique co‐localizing proteins each supporting the sequential nature of their action.^52^

Another interesting observation is that only few proteins co‐localized to COPII‐positive structures (ie, 12, 2 of which, RFT1 and YIP1, were shared with the Cop1 and Anp1 compartments, respectively). This is different from COPI‐positive structures that had a much larger (86) protein roster. This may either reflect the fact that N′ tagging of COPII vesicle proteins may affect their sorting or that the actual COPII vesicles are too small or short lived to be captured by light microscopy. It could also just be a technical short‐come of the marker protein we chose to use. Indeed, the COPII marker used showed very small and dim punctate signals. In support of the latter, many proteins that were annotated as residing in COPII vesicles do not co‐localize with its marker and instead localize to COPI‐positive structures, perhaps during their retrieval to the ER.

Many proteins that co‐localized with the COPI marker were also found in other Golgi compartments. Two distinct groups of COPI proteins could be observed—those that also localize to the Anp1 compartment (38), and those that are shared with the Sec7‐Chc1 compartment (30). This supports the existence of 2 separate roles for the COPI coat as facilitating cargo retrieval from the Golgi to the ER on the one hand and intra‐Golgi traffic on the other.^53^, ^54^, ^55^ Surprisingly, we found 13 proteins that co‐localized with both COPI and the early endosome marker Snf7 suggesting that even early endocytic vesicles can still be served by COPI retrograde traffic as suggested by previous studies.^56^, ^57^

Our analysis included 69 proteins that co‐localize with the early endosome marker Snf7. Several of these proteins were also localized to the earlier compartments in the secretory system including Cop1‐positive structures and/or the Golgi compartments marked by Sec7 and Chc1. Only 3 proteins were also localized to the Anp1 compartment (Ktr3, Gdt1 and Tmn2). Another separate group of Snf7 co‐localized proteins also co‐localized with the late endosome marker Vam6 (30). This group of proteins is dedicated to transporting proteins to the vacuole—either for degradation or processing as their final fate. The extensive overlap between co‐localizing proteins with Snf7 and Vam6 suggests that they mark at least one shared organelle.

Altogether, the systematic analysis of N′ GFP‐tagged endomembrane system proteins has revealed several preliminary yet novel insights regarding the structure of the endomembrane system. The accurate imaging of several uncharacterized proteins will now hopefully lead to a better understanding of their functions. Finally, we hope that the integration of the data from this study with what is already known about the different organelles of the endomembrane system will add to the emerging picture of how a third of the cells proteome is targeted to its precise cellular destination.

## 4 MATERIAL AND METHODS

### 4.1 Marker mCherry yeast strain construction

Marker strains were constructed on the background of an Synthetic genetic array (SGA) compatible query strain (YMS721) of mating type Alpha.^58^ In these strains we C terminally tagged marker proteins by genomically introducing a NAT::mCherry‐ADHtr cassette in place of the stop codon for the following genes: Anp1, Chc1 and Sec7 to mark 3 Golgi compartments, Cop1 to mark COPI vesicles, Sec13 to mark COPII vesicles and Snf7 to mark early endosomes. Similar tagging was done for Nvj2 on the GFP‐Lam5 and GFP‐Lam5/Δ*nvj1* strains. For Vam6 we used N‐terminal tagging as C‐terminal tagging ruins the functionality of the protein (using A NAT::TEF2pr‐mCherry cassette). This was done to mark late endosomes. In order to mark the ER with Cyan fluorescent protein (CFP) we used the pRs315‐PGK‐CFP‐HDEL‐LEU2 plasmid. All genetic yeast transformations were carried out via a standard PEG‐LiAc protocol.^59^

### 4.2 Yeast strains assembly and manipulation

All N′ GFP‐tagged yeast strains were cherry picked from the SWAT‐SP‐GFP (that included the SP of the Kar2 protein) and SWAT‐GFP collections, both including the constitutive, medium strength promotor of the *sp*NOP1 gene and are of mating type A.^15^ Only strains that were initially annotated with “punctate” as one of their localizations were manually picked and rearrayed into 384‐well format (Table S1). We conducted automated strain maintenance and manipulation using a RoToR benchtop colony arrayer^60^ (Singer Instruments, Roadwater, Watchet, UK). We performed automated mating and selection procedures^58^ for mating of the N′ GFP‐tagged yeast strains array with mCherry‐tagged marker strains. The final crossed array included both the N′ GFP tag and an mCherry marker in diploid strains.

### 4.3 High‐throughput microscopy

High‐content screening of strain collections was performed using an automated microscopy setup (ScanR system, Olympus, Waltham, Massachusetts, USA) as previously described.^13^ Images were acquired using a ×60 air lens for GFP (excitation, 490/20 nm; emission, 535/50 nm), mCherry (excitation, 572/35 nm; emission, 632/60 nm), CFP (excitation, 402/15 nm; emission, 455/50 nm) and brightfield channels. For localization assignments, images were reviewed manually using the ImageJ software (U. S. National Institutes of Health, Bethesda, Maryland, USA) and the relationship between the GFP and mCherry signals was annotated as either full, partial or no co‐localization.

### 4.4 Data processing

Comparison of the subcellular‐localization annotations of the SWAT‐GFP libraries (with the generic promoter and SP) with those of the C′‐tag library^6^ was performed using the following annotations: “Same” was assigned when the N′ annotation corresponded exactly to a C′ one. “New” was assigned if the C′ localization was classified as below threshold or ambiguous, or if no assignment existed. All other cases were classified as “Different.”

### 4.5 GFP‐based affinity purifications

Yeast cells were grown to early‐stationary phase. For isolation of GFP‐tagged coatomer (Figure 2B), the cell pellet was flash frozen in liquid nitrogen, crushed by hand and resuspended in lysis buffer (10 mM sodium phosphate, pH 7.4, 150 mM NaCl, 1 mM Ethylenediaminetetraacetic acid (EDTA), 1 mM DTT and supplemented with protease inhibitors), dounce homogenized (12 strokes) and centrifuged at 100 000*g* for 30 minutes. The pellet was resuspended in 500 μL of solubilization buffer (1% TX‐100, 150 mM NaCl, 25 mM HEPES pH 7.4, supplemented with protease inhibitors). The extract was centrifuged at 50 000*g* for 15 minutes, diluted 1:4 and incubated with Chromotek GFP‐Trap magnetic particles for 60 minutes. Following 5 washes the affinity matrix was eluted with 1× SDS‐Sample buffer containing 100 mM DTT. For GFP‐based pulldown experiments (Figure 2C), cells were flash frozen, crushed by hand and resuspended in lysis buffer (25 mM Tris pH 7.5, 50 mM KCl, 10 mM MgCl_2_, 5% Glycerol, 1% IGEPAL and 1 mM DTT and supplemented with protease inhibitors). The samples were centrifuged at 500*g* for 10 minutes, treated with 50 U benzonase for 30 minutes and centrifuged at 5000*g* for 10 minutes. The extract was incubated with Chromotek GFP‐Trap agarose particles for 60 minutes. Following 4 washes (with 50 mM Tris pH 7.5, 150 mM NaCl, 5% Glycerol, 0.05% IGEPAL and 1 mM DTT) the affinity matrix was eluted with 1×‐SDS‐sample buffer containing 100 mM DTT.

## Supporting information


**Editorial Process**

**Figure S1** Golgi protein markers overlap profile. Anp1, Sec7 and Chc1 mCherry‐tagged against their N′ GFP forms. All scale bars are 5 μm.
**Figure S2** N′ tagged proteins that fully co‐localize with Anp1‐mCherry (Golgi). All scale bars are 5 μm.
**Figure S3** N′ tagged proteins that fully co‐localize with Sec7‐mCherry (Golgi). All scale bars are 5 μm.
**Figure S4** N′ tagged proteins that fully co‐localize with Chc1‐mCherry (Golgi). All scale bars are 5 μm.
**Figure S5** N′ tagged proteins that fully co‐localize with Cop1‐mCherry (COPI). All scale bars are 5 μm.
**Figure S6** N′ tagged proteins that fully co‐localize with Snf7‐mCherry (endosome). All scale bars are 5 μm.
**Figure S7** N′ tagged proteins that fully co‐localize with mCherry‐Vam6 (late endosome) or Sec13‐mCherry (COPII). All scale bars are 5 μm.
**Figure S8** Newly found proteins residing in the compartments of the secretory system. N′ tagged proteins without a previously described cellular localization or function were found to reside within one or more compartments by co‐localization with an mCherry‐tagged protein markers. All scale bars are 5 μm.
**Figure S9** Tagging at the 2 termini has an effect on steady state localization. Different cellular localization for N′ tagged proteins. Shown are proteins that co‐localized with secretory system compartment markers (mCherry) only when they are tagged with GFP at their N′. All scale bars are 5 μm.

**Table S1** List of N′ GFP‐tagged proteins showing a punctate localization.

**Table S2** Annotations for N′ GFP‐tagged proteins showing co‐localization with endomembrane compartment markers.
