# Supplementary material for: Toolbox: Creating a systematic database of secretory pathway proteins uncovers new cargo for COPI
Source: Traffic. 2018 Apr 10;19(5):370–9. doi: 10.1111/tra.12560 (PMC5947560; doi:10.1111/tra.12560)
Supplement: Supplementary file 1 — Editorial Process [file TRA-19-370-s001.pdf]

Title:	**Toolbox: Creating a systematic database of secretory pathway proteins uncovers new cargo for COPI**
Authors:	Uri Weill, Eric C. Arakel, Omer Goldmann, Matan Golan, Silvia Chuartzman, Sean Munro , Blanche Schwappach & Maya Schuldiner
Article Type:	Toolbox

Monitoring Editor	Christian Ungermann
Date Submitted	16 November 2017
Date for Decision 1	20 December 2017
Date Resubmitted	17 February 2018
Date for Decision 2	20 February 2018
Accepted	25 February 2018

Decision and Reviews

Dear Maya,

Thank you for submitting your manuscript "Toolbox: Creating a systematic database of secretory pathway proteins uncovers new cargo for COPI" for consideration for publication as a Toolbox paper in Traffic. I asked two colleagues who are experts in the field to review the paper and their verbatim comments are appended below. The referees share the view that the work presented in this paper is a very useful resource for the community. Both referees have made recommendations to correct and improve the presentation that you will need to address before this paper can be accepted. The referees have clearly outlined their concerns so I will not reiterate these here.

Although I cannot accept your manuscript for publication at this point, I believe that you will be able to address the referees' concerns and I look forward to receiving your revised manuscript. To expedite handling when you resubmit please be sure to include a response outlining how you have addressed each of the referees' concerns.

Sincerely,

Christian

Christian Ungermann, Ph.D.
Associate Editor

____

Referee's Comments to the Authors

Referee: 1

Comments to the Author
In this study, Weill et al provide a more complete localization atlas of endomembrane proteins in yeast by imaging live cells expressing GFP-tagged versions of these proteins together with known marker proteins. In total, 231 N-terminally GFP-tagged proteins were systematically monitored for co-localization with multiple ER, Golgi and endosomal makers. The report provides new insights on several of these proteins and will be a valuable resource for investigators across disciplines. I have only a few suggestions to strengthen this important work.

1. On page 4, defining full co-localization – where the two patterns were "exactly the same", might not be the best wording. There are several images where there is a high level of co-localization but also occasional separation of markers. Perhaps this could be defined as the patterns were "mostly the same" or "largely the same".

2. In the legend to Fig. 2, please indicate if panel 2B is a protein stain or immunoblot with anti-coatomer.

3. In Fig. 3B and Fig. 3D, are there better images or can the brightness for GFP-Atg9 and GFP-Rsp5 be reduced for better resolution? It might be the way these images converted to the pdf file but in many cells it is difficult to see distinct puncta for GFP-Atg9 and GFP-Rsp5 to make co-localization by overlay with markers apparent.

4. Regarding the description of Atg9 on page 7, if Atg9 cycles between endosomes and late-Golgi, would one expect co-localization with Cop1? In table 2, only Sec21 shows partial co-localization with Sec7, a standard late-Golgi marker. Or is Atg9 thought to be in a new COPI coated structure? It might help to clarify the thinking here. Possibly related, a recently published paper in eLife (Xu et al., 2017: PMC5663479) suggests that a fraction of COPI binds polyubiquitinated cargo in endosomes for delivery to late-Golgi.

5. On page 7, there is mention of "a specific interaction between Lam5 and the Golgi protein Arl1 (data not shown)…" It would be

informative to state how this interaction was experimentally detected. Also, regarding the Lam5 localization pattern in Fig. 3F, this seems unusual for typical ER-localized proteins and it might be good to comment on this point.

Referee: 2

Comments to the Author
This “Toolbox” submission re-evaluates an existing yeast GFP fusion library to determine the localization of several hundred N-terminally-tagged proteins in the secretory and endocytic compartments. Among the new insights are the identification of Lam5 as a putative marker for ER/Golgi contact sizes, and the identification of novel proteins that colocalize with COPI and are potential new cargo for this coat.

In general, this project was carefully done and will be a useful resource. For this type of survey, there is a limit to the depth and quality of the analysis that can be expected, but based on the impressive utility of the earlier C-terminal GFP tagging library for yeast, t is likely that the research community will appreciate the information. I have just a few comments about the interpretations:

1) Recent work indicates that yeast does not actually have distinct early and late endosomes. This result is not yet published, but a preprint is available as a Sneak Peek from Developmental Cell:

https://www.mendeley.com/sneak-peek/cellpress/#f3ece503-e335-4d37-a88d-cfcad544e123

It seems likely that Snf7 labels late endosomes, and Vam6 labels the same late endosomes together with vacuoles.

2) The Lam5 discussion should reference the recent description of Nvj2 as a tether that generates ER-Golgi contact sites in yeast:

https://www.ncbi.nlm.nih.gov/pubmed/28011845

A colocalization test with Nvj2 would be useful.

3) For the various Golgi proteins, cisternal maturation complicates the analysis because different “compartments” may actually represent different stages in the pathway. This temporal issue has been documented for proteins such as Sec7 and Chc1 and should be acknowledged:

https://www.ncbi.nlm.nih.gov/pubmed/22344030

Moreover, the arrival and departure times of COPI versus other Golgi proteins, and the corresponding colocalization values, have been described in yet another paper that should be cited:

https://www.ncbi.nlm.nih.gov/pubmed/26906739

4) Is it safe to add an N-terminal GFP tag to type II Golgi membrane proteins? My impression is that in mammalian cells, such fusions can perturb localization.

Author Rebuttal

Point by point response to reviewer’s concerns

Referee: 1

1. *On page 4, defining full co-localization – where the two patterns were “exactly the same”, might not be the best wording. There are several images where there is a high level of co- localization but also occasional separation of markers. Perhaps this could be defined as the patterns were “mostly the same” or “largely the same”.*

We thank the reviewer for this accurate comment. The text has now been changed to define co- localization as “where the signals from the two channels show high degree of overlap”. Also, in Figure 1 we have removed the word “Full” and left simply “co-localization”.

2. *In the legend to Fig. 2, please indicate if panel 2B is a protein stain or immunoblot with anti- coatomer.*

The figure legend has now been updated to denote that in panel 2B “All Western blots were detected with an antibody raised against coatomer”.

3. *In Fig. 3B and Fig. 3D, are there better images or can the brightness for GFP-Atg9 and GFP- Rsp5 be reduced for*

better resolution? It might be the way these images converted to the pdf file but in many cells it is difficult to see distinct puncta for GFP-Atg9 and GFP-Rsp5 to make co- localization by overlay with markers apparent.

The figures have now been exchanged to ones with better capacity to visualized distinct puncta.

4. Regarding the description of Atg9 on page 7, if Atg9 cycles between endosomes and late- Golgi, would one expect co-localization with Cop1? In table 2, only Sec21 shows partial co- localization with Sec7, a standard late-Golgi marker. Or is Atg9 thought to be in a new COPI coated structure? It might help to clarify the thinking here. Possibly related, a recently published paper in eLife (Xu et al., 2017: PMC5663479) suggests that a fraction of COPI binds polyubiquitinated cargo in endosomes for delivery to late-Golgi.

The text has now been changed to stress that we suggest that Atg9 is indeed in a COPI coated structure. However we also add that "It has also recently been published that a fraction of COPI binds polyubiquitinated cargo in endosomes for delivery to late-Golgi [35]. However, for technical reasons, we could not assay co-localization of GFP-Atg9 with the Sec7-mCherry, Snf7-mCherry or Vam6-mCherry markers".

5. On page 7, there is mention of "a specific interaction between Lam5 and the Golgi protein Arl1 (data not shown)…" It would be informative to state how this interaction was experimentally detected. Also, regarding the Lam5 localization pattern in Fig. 3F, this seems unusual for typical ER-localized proteins and it might be good to comment on this point.

The text has now been changed to state that the interaction was found by a co-ip and we have added a comment about Lam5 not being distributed evenly on the ER membrane like a regular membrane protein but rather as a contact site protein is expected to be, in discrete punctate structures on the surface of the ER.

Referee: 2

1) Recent work indicates that yeast does not actually have distinct early and late endosomes. This result is not yet published, but a preprint is available as a Sneak Peek from Developmental Cell:

https://www.mendeley.com/sneak-peek/cellpress/#f3ece503-e335-4d37-a88d-cfcad544e123

Thank you for the insightful comment. A discussion about how much our data supports a view for distinct endosome compartments has been added as has the above reference.

2) It seems likely that Snf7 labels late endosomes, and Vam6 labels the same late endosomes together with vacuoles.

Our data shows that there isn't completely overlap between these two markers suggesting that they do mark some structures that are unique. However we have added a comment in the text that these markers have an extensive overlap suggesting that that they mark at least one shared organelle.

3) The Lam5 discussion should reference the recent description of Nvj2 as a tether that generates ER-Golgi contact sites in yeast: https://www.ncbi.nlm.nih.gov/pubmed/28011845. A colocalization test with Nvj2 would be useful.

We thank the reviewer for this important point and have now added reference the recent description of Nvj2 as a tether that generates ER-Golgi contact sites in yeast. We further checked co-localization between GFP-Lam5 and Nvj2-mCherry and found that in the background of a deletion in ∆nvj1 where Nvj2 goes only to the ER/Golgi contacts there is indeed co- localization between the two proteins as shown in updated figure 2E. Proving now, unequivocally that Lam5 is an ER/Golgi contact site protein. We also discuss that since Lam5 is

present at these contacts before Nvj2 moves to them, it may seed Nvj2 movement to these contacts.

4) For the various Golgi proteins, cisternal maturation complicates the analysis because different "compartments" may actually represent different stages in the pathway. This temporal issue has been documented for proteins such as Sec7 and Chc1 and should be acknowledged:

https://www.ncbi.nlm.nih.gov/pubmed/22344030

Moreover, the arrival and departure times of COPI versus other Golgi proteins, and the corresponding colocalization values, have been described in yet another paper that should be cited:

https://www.ncbi.nlm.nih.gov/pubmed/26906739

We thank the reviewer for bringing up these references which we have now added to the text in an extended discussion about Golgi cisternal maturation.

4) Is it safe to add an N-terminal GFP tag to type II Golgi membrane proteins? My impression is that in mammalian cells, such fusions can perturb localization.

This is a very important point – in general adding a large tag as GFP can be deleterious to protein folding and localization. Hence, any individual protein studies must demonstrate that the GFP fusion (N or C) covers for the function of the protein. In this case, we do not believe that our N' tag created a specific problem. The reason is that Type 2 proteins that have their N' facing the cytosol should be fine to tag as long as they are not from the "signal anchor" type – hence no cytosolic amino acids are expected on the cytosolic side – in which the cytosolic GFP could dramatically alter the structure of the protein.

To see how many such signal anchor proteins we have in our data we have taken all of the proteins that have a Golgi co-localization and checked if any of them are predicted to be type 2 signal anchor (ie, single TMD at the very N') and find that only 7 proteins have a transmembrane domain less than 40 aa away from the start codon. Out of them 6 are known proteins that were in the right location (co localizing with Anp1/the rest of their complex) according to the literature: Ktr5, Mnn9, Och1, Anp1, Ktr7 and Mnn11. We therefore assume that the additional protein Ykl063c is also in the right local.

Decision and Reviews

Dear Dr. Schuldiner,

Christian Ungermann asked me to write to you on his behalf to thank you for making the changes in your manuscript "Toolbox: Creating a systematic database of secretory pathway proteins uncovers new cargo for COPI". You have now addressed the concerns raised previously and this paper is now accepted for publication in Traffic.

There are a few things I will need from you before I can get this paper into press.
1) I am not convinced that the abstract figure you suggested does a good job of conveying the main points of your paper. Do you have an alternative figure that you can use that more clearly describes your approach and the results from your paper?
2) Please send the abstract figure as a stand alone file, and send a final version of the text to be used for copyediting.
3) Please send all of the files and forms detailed here and below through the online system.

Thank you for sending this work to Traffic.

Best wishes,
Lisa Hannan
