# Supplementary material for: Toolbox: Creating a systematic database of secretory pathway proteins uncovers new cargo for COPI
Source: Traffic. 2018 Apr 10;19(5):370–9. doi: 10.1111/tra.12560 (PMC5947560; doi:10.1111/tra.12560)
Supplement: Supplementary file 2 — Figure S1 Golgi protein markers overlap profile. Anp1, Sec7 and Chc1 mCherry‐tagged against their N′ GFP forms. All scale bars are 5 μm. Figure S2 N′ tagged proteins that fully co‐localize with Anp1‐mCherry (Golgi). All scale bars are 5 μm. Figure S3 N′ tagged proteins that fully co‐localize with Sec7‐mCherry (Golgi). All scale bars are 5 μm. Figure S4 N′ tagged proteins that fully co‐localize with Chc1‐mCherry (Golgi). All scale bars are 5 μm. Figure S5 N′ tagged proteins that fully co‐localize with Cop1‐mCherry (COPI). All scale bars are 5 μm. Figure S6 N′ tagged proteins that fully co‐localize with Snf7‐mCherry (endosome). All scale bars are 5 μm. Figure S7 N′ tagged proteins that fully co‐localize with mCherry‐Vam6 (late endosome) or Sec13‐mCherry (COPII). All scale bars are 5 μm. Figure S8 Newly found proteins residing in the compartments of the secretory system. N′ tagged proteins without a previously described cellular localization or function were found to reside within one or more compartments by co‐localization with an mCherry‐tagged protein markers. All scale bars are 5 μm. Figure S9 Tagging at the 2 termini has an effect on steady state localization. Different cellular localization for N′ tagged proteins. Shown are proteins that co‐localized with secretory system compartment markers (mCherry) only when they are tagged with GFP at their N′. All scale bars are 5 μm. [file TRA-19-370-s002.pdf]

Toolbox: Creating a systematic database of secretory pathway proteins uncovers new cargo for COPI

Uri Weill[1#], Eric C. Arakel[2#], Omer Goldmann[1], Matan Golan[1], Silvia Chuartzman[1], Sean Munro[3], Blanche Schwappach[2*] & Maya Schuldiner[1*]

1. Department of Molecular Genetics, Weizmann Institute of Science, Rehovot 7610001, Israel
2. Universitätsmedizin Göttingen Institut für Molekularbiologie Humboldtallee 23 D-37073 Göttingen and Max-Planck Institute for Biophysical Chemistry, 37077 Göttingen, Germany.
3. MRC Laboratory of Molecular Biology, Francis Crick Avenue, Cambridge CB2 0QH, UK.

[#] These authors contributed equally to this work

[*] Correspondence should be addressed to:

blanche.schwappach@med.uni-goettingen.de;

Blanche Schwappach Office Tel: +49 551 395961

Blanche Schwappach Fax: +49 551 395960

maya.schuldiner@weizmann.ac.il

Maya Schuldiner Office Tel: +972-8-934-6346

Maya Schuldiner Lab Tel: +972-8-934-6460

Maya Schuldiner Fax: +972-8-934-6373

Supplementary materials:

Supplementary figures:

Supplementary figure 1: Golgi protein markers overlap profile

Anp1, Sec7 and Chc1 mCherry tagged against their N'GFP forms. All scale bars are 5µm.

Supplementary figure 2: N' tagged proteins that fully co-localize with Anp1-mCherry (Golgi)

All scale bars are 5µm.

Supplementary figure 3: N' tagged proteins that fully co-localize with Sec7-mCherry (Golgi)

All scale bars are 5µm.

Supplementary figure 4: N' tagged proteins that fully co-localize with Chc1-mCherry (Golgi)

All scale bars are 5µm.

Supplementary figure 5: N' tagged proteins that fully co-localize with Cop1-mCherry (COPI)

All scale bars are 5µm.

Supplementary figure 6: N' tagged proteins that fully co-localize with Snf7-mCherry (endosome)

All scale bars are 5µm.

Supplementary figure 7: N' tagged proteins that fully co-localize with mCherry-Vam6 (late endosome) or Sec13-mCherry (COPII)

All scale bars are 5µm.

Supplementary figure 8: Newly found proteins residing in the compartments of the secretory system

N' tagged proteins without a previously described cellular localization or function were found to reside within one or more compartments by co-localization with an mCherry tagged protein markers. All scale bars are 5µm.

Supplementary figure 9: Tagging at the two termini has an effect on steady state localization

Different cellular localization for N' tagged proteins. Shown are proteins that co-localized with secretory system compartment markers (mCherry) only when they are tagged with GFP at their N'. All scale bars are 5µm.

Supplementary Tables:

Supplementary Table 1: List of N' GFP tagged proteins showing a punctate localization.

Supplementary Table 2: Annotations for N' GFP tagged proteins showing co-localization with endomembrane compartment markers.

Sup. Figure 1

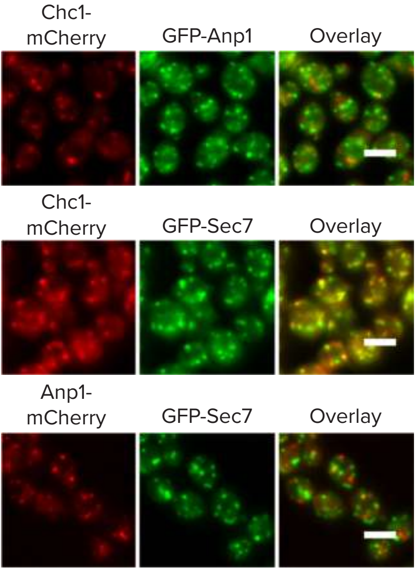

Chc1-mCherry
GFP-Anp1
Overlay
Chc1-mCherry
GFP-Sec7
Overlay
Anp1-mCherry
GFP-Sec7
Overlay

Sup. Figure 2

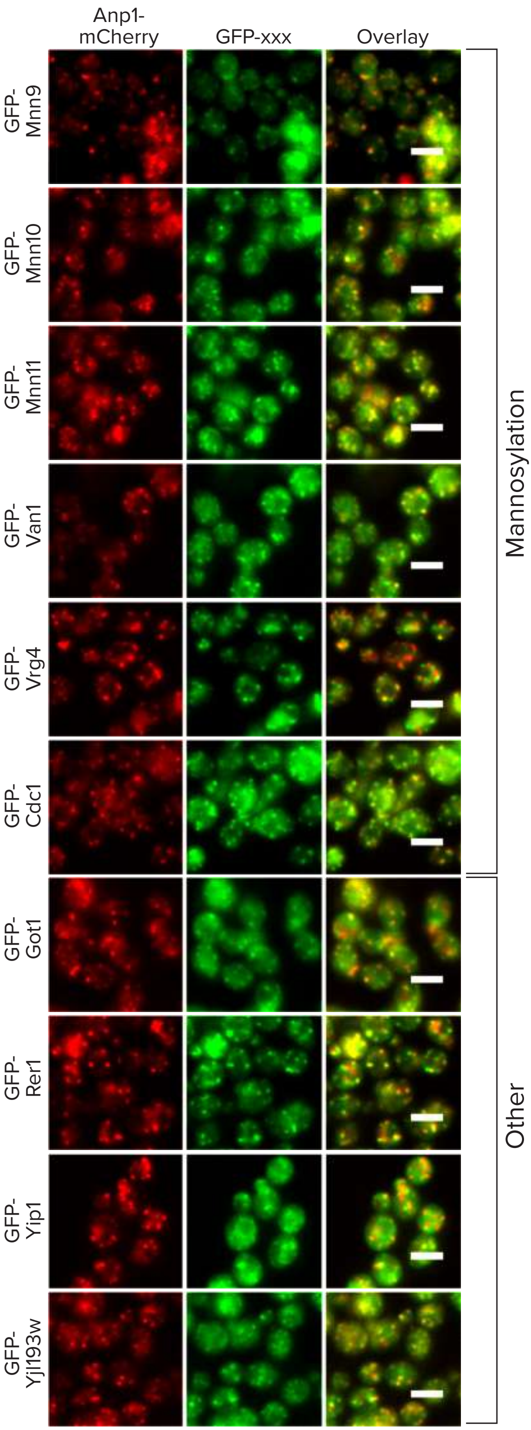
Anp1-mCherry
GFP-xxx
Overlay
GFP-Mnn9
GFP-Mnn10
GFP-Mnn11
GFP-Van1
GFP-Vrg4
GFP-Cdc1
GFP-Got1
GFP-Rer1
GFP-Yip1
GFP-Yjl193w
Mannosylation
Other

Sup. Figure 3

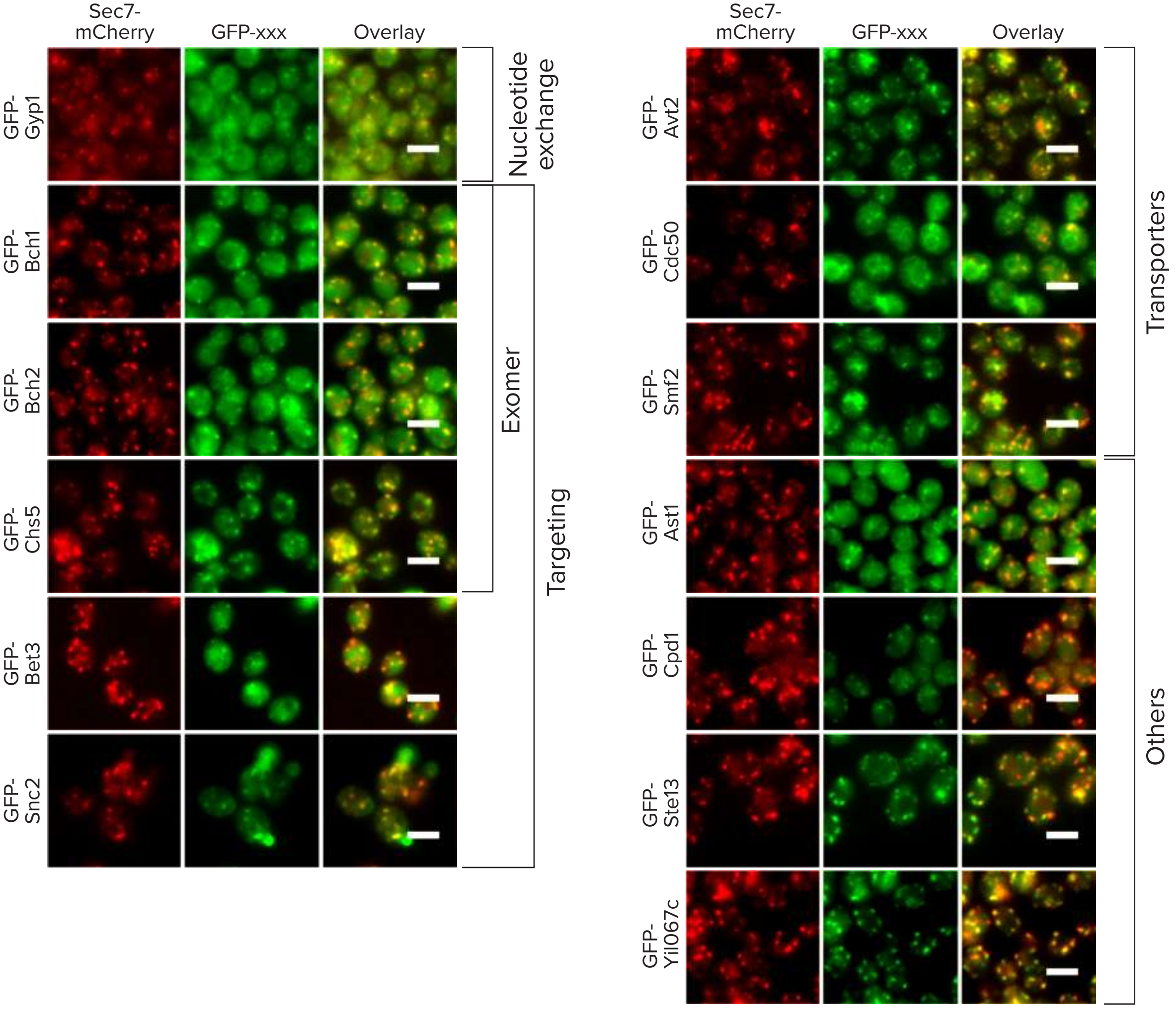
Sec7-mCherry
GFP-xxx
Overlay
GFP-Gyp1
GFP-Bch1
GFP-Bch2
GFP-Chs5
GFP-Bet3
GFP-Snc2
Nucleotide exchange
Exomer
Targeting
Sec7-mCherry
GFP-xxx
Overlay
GFP-Avt2
GFP-Cdc50
GFP-Smf2
GFP-Ast1
GFP-Cpd1
GFP-Ste13
GFP-Yil067c
Transporters
Others

Sup. Figure 4

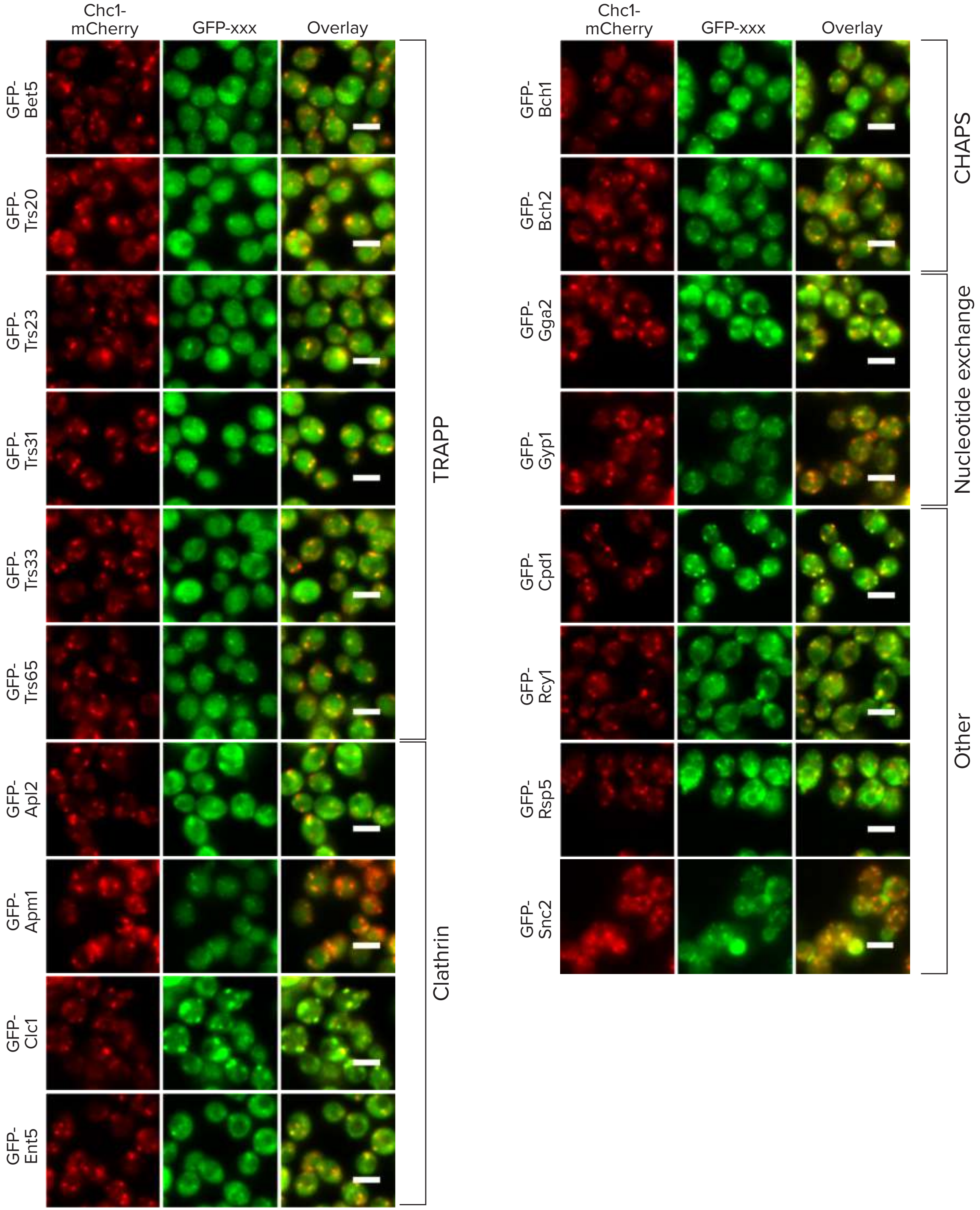
Chc1-mCherry
GFP-xxx
Overlay
GFP-Bet5
GFP-Trs20
GFP-Trs23
GFP-Trs31
GFP-Trs33
GFP-Trs65
TRAPP
GFP-Apl2
GFP-Apm1
GFP-Clc1
GFP-Ent5
Clathrin
GFP-Bch1
GFP-Bch2
CHAPS
GFP-Gga2
GFP-Gyp1
Nucleotide exchange
GFP-Cpd1
GFP-Rcy1
GFP-Rsp5
GFP-Snc2
Other

Sup. Figure 5

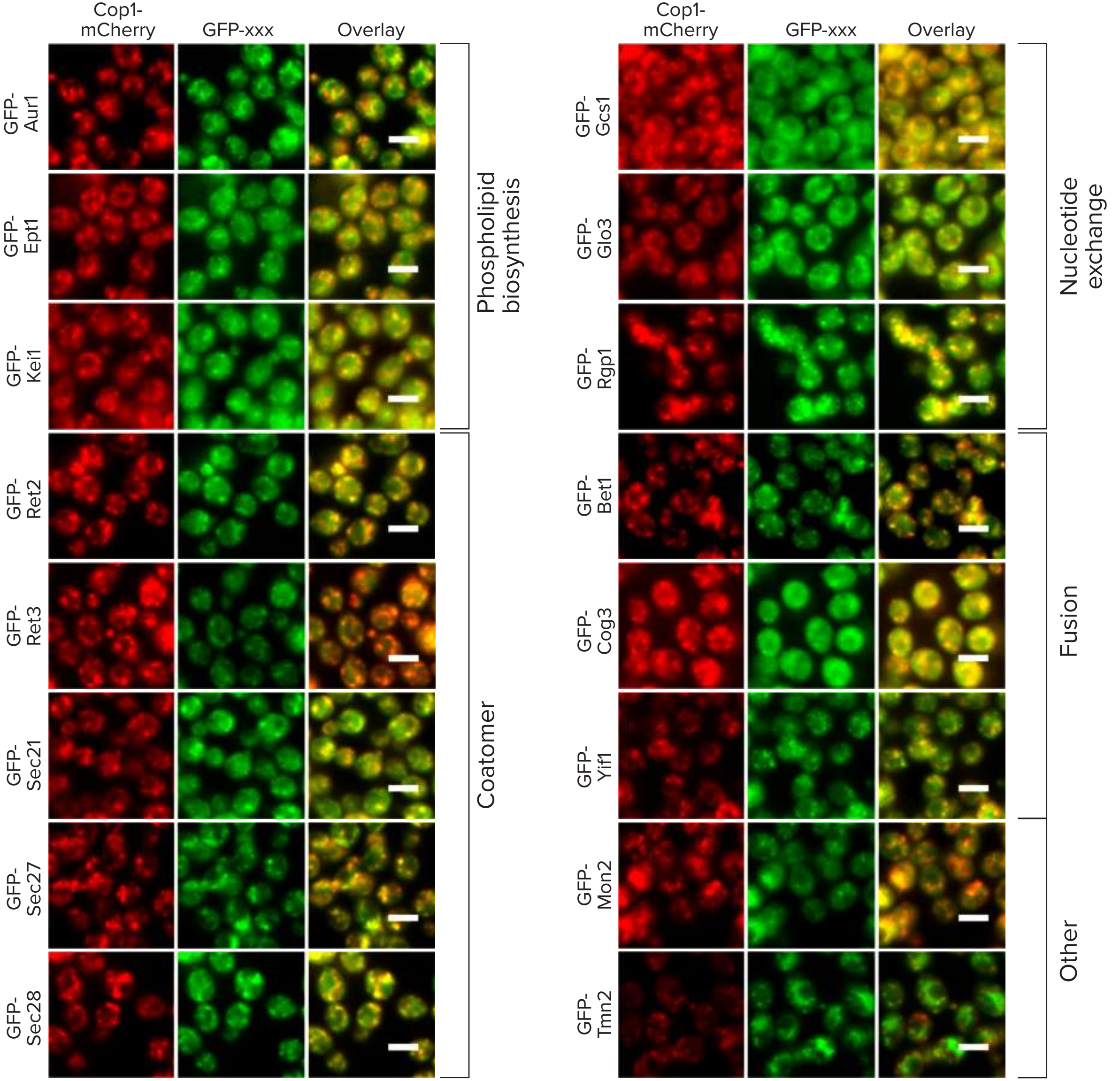

Cop1-mCherry
GFP-xxx
Overlay
GFP-Aur1
GFP-Ept1
GFP-Kei1
GFP-Ret2
GFP-Ret3
GFP-Sec21
GFP-Sec27
GFP-Sec28
Phospholipid biosynthesis
Coatomer
Cop1-mCherry
GFP-xxx
Overlay
GFP-Gcs1
GFP-Glo3
GFP-Rgp1
GFP-Bet1
GFP-Cog3
GFP-Yif1
GFP-Mon2
GFP-Tmn2
Nucleotide exchange
Fusion
Other

Sup. Figure 6

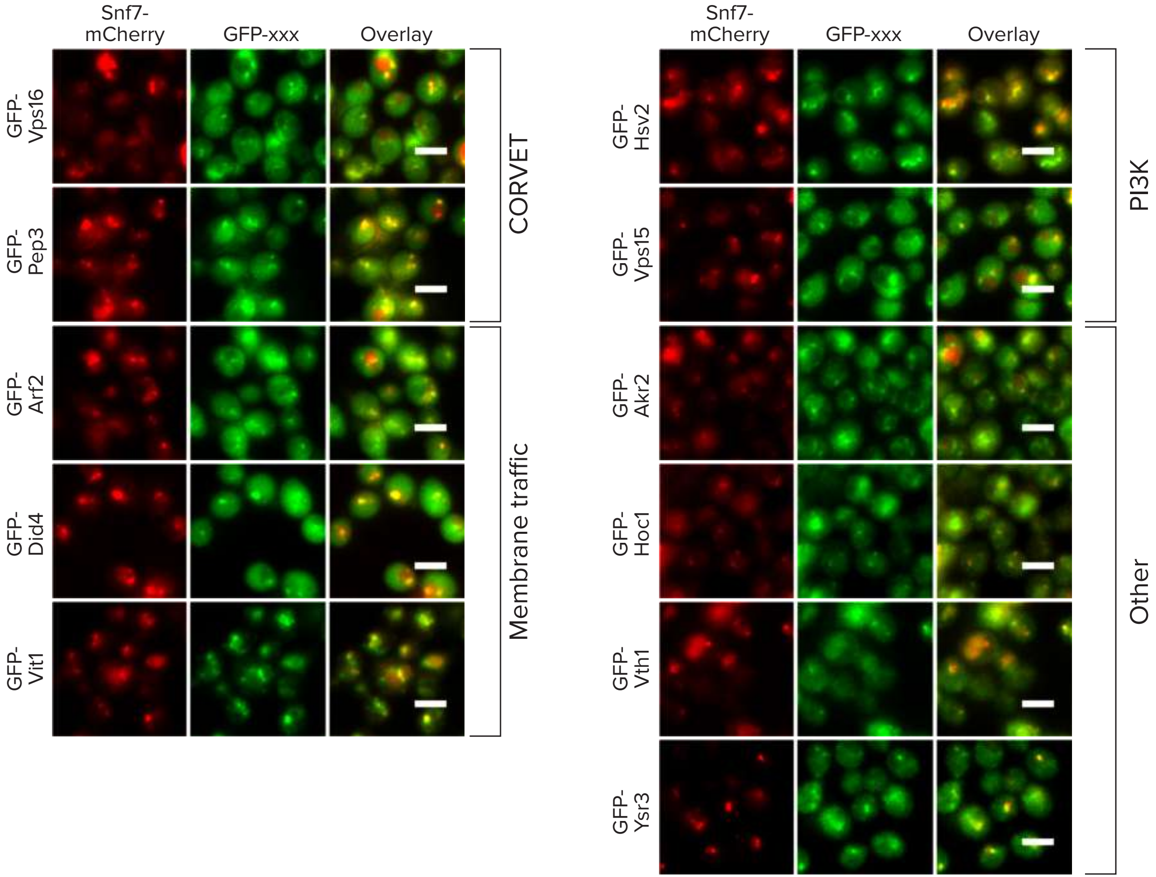
Snf7-mCherry
GFP-xxx
Overlay
GFP-Vps16
GFP-Pep3
GFP-Arf2
GFP-Did4
GFP-Vit1
CORVET
Membrane traffic
Snf7-mCherry
GFP-xxx
Overlay
GFP-Hsv2
GFP-Vps15
GFP-Akr2
GFP-Hoc1
GFP-Vth1
GFP-Ysr3
PI3K
Other

Sup. Figure 7

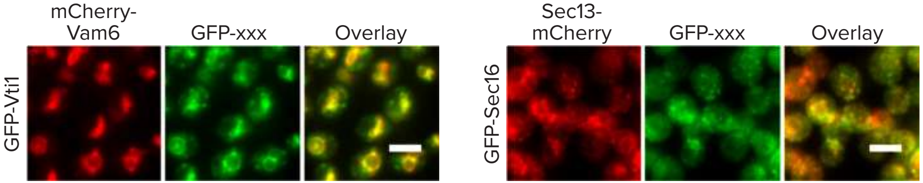

mCherry-Vam6
GFP-xxx
Overlay
GFP-Vti1
Sec13-mCherry
GFP-xxx
Overlay
GFP-Sec16

Sup. Figure 8

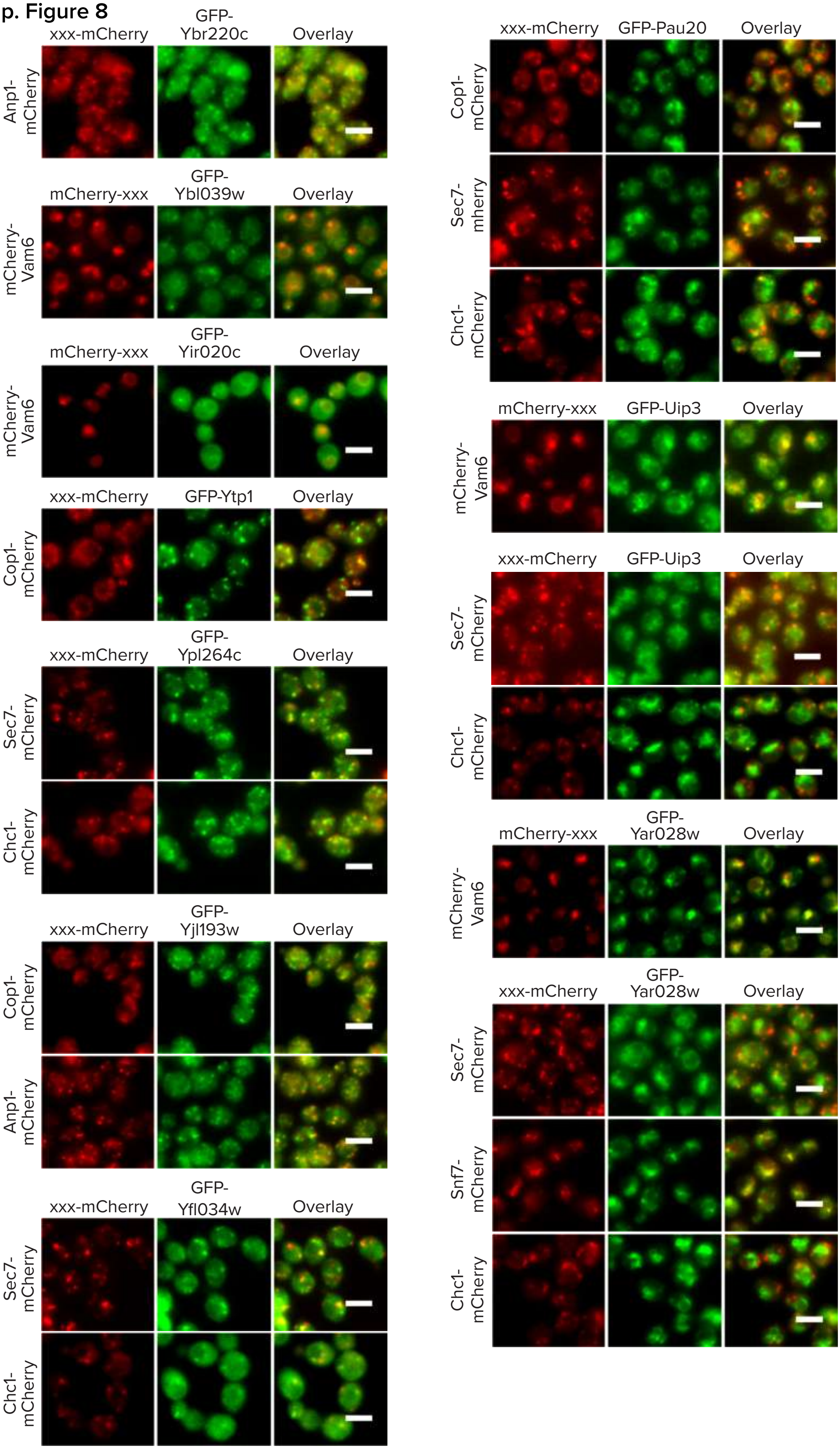
xxx-mCherry
GFP-Ybr220c
Overlay
Anp1-mCherry
mCherry-xxx
GFP-Ybl039w
Overlay
mCherry-Vam6
mCherry-xxx
GFP-Yir020c
Overlay
mCherry-Vam6
xxx-mCherry
GFP-Ytp1
Overlay
Cop1-mCherry
xxx-mCherry
GFP-Ypl264c
Overlay
Sec7-mCherry
Chc1-mCherry
xxx-mCherry
GFP-Yjl193w
Overlay
Cop1-mCherry
Anp1-mCherry
xxx-mCherry
GFP-Yfl034w
Overlay
Sec7-mCherry
Chc1-mCherry
xxx-mCherry
GFP-Pau20
Overlay
Cop1-mCherry
Sec7-mherry
Chc1-mCherry
mCherry-xxx
GFP-Uip3
Overlay
mCherry-Vam6
xxx-mCherry
GFP-Uip3
Overlay
Sec7-mCherry
Chc1-mCherry
mCherry-xxx
GFP-Yar028w
Overlay
mCherry-Vam6
xxx-mCherry
GFP-Yar028w
Overlay
Sec7-mCherry
Snf7-mCherry
Chc1-mCherry

Sup. Figure 9

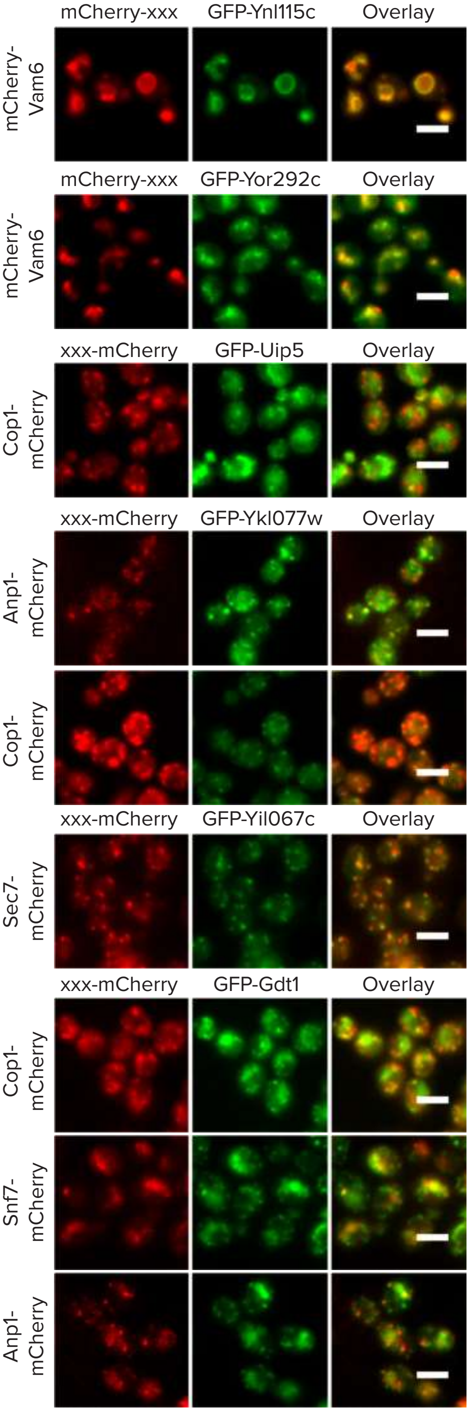
mCherry-xxx
GFP-Ynl115c
Overlay
mCherry-Vam6
mCherry-xxx
GFP-Yor292c
Overlay
mCherry-Vam6
xxx-mCherry
GFP-Uip5
Overlay
Cop1-mCherry
xxx-mCherry
GFP-Ykl077w
Overlay
Anp1-mCherry
Cop1-mCherry
xxx-mCherry
GFP-Yil067c
Overlay
Sec7-mCherry
xxx-mCherry
GFP-Gdt1
Overlay
Cop1-mCherry
Snf7-mCherry
Anp1-mCherry
